# Hepatitis A vaccination and its public-health impact in routine prevention and outbreak control: a systematic review and meta-analysis

**DOI:** 10.1016/j.eclinm.2026.104144

**Published:** 2026-08-11

**Authors:** Jana Malinová, Marek Petráš, Jiří Sýkora, Pavel Dlouhý, Jozef Rosina, Ivana Králová Lesná

**Affiliations:** a Department of Occupational and Travel Medicine, Third Faculty of Medicine, Charles University, Prague, Czech Republic; b Královské Vinohrady University Hospital, Prague, Czech Republic; c Department of Epidemiology and Biostatistics, Third Faculty of Medicine, Charles University, Prague, Czech Republic; d Department of Hygiene, Third Faculty of Medicine, Charles University, Prague, Czech Republic; e Department of Medical Biophysics and Informatics, Third Faculty of Medicine, Charles University, Prague, Czech Republic; f Department of Health Care and Population Protection, Faculty of Biomedical Engineering, Czech Technical University, Prague, Czech Republic; g Laboratory for Atherosclerosis Research, Centre for Experimental Medicine, Institute for Clinical and Experimental Medicine, Prague, Czech Republic; h Department of Anesthesia and Intensive Medicine, First Faculty of Medicine, Charles University and Military University Hospital, Prague, Czech Republic

**Keywords:** HAV, Vaccine effectiveness, Vaccine impact, Pre-exposure vaccination, Post-exposure vaccination

## Abstract

**Background:**

Hepatitis A vaccination is used for routine prevention and outbreak control, but its effectiveness and population-level impact have not been comprehensively synthesised. We aimed to assess vaccine effectiveness, certainty of evidence, and population-level impact.

**Methods:**

In this systematic review and meta-analysis, we searched MEDLINE, Embase, Biological Abstracts, and CENTRAL for studies published from 1st January 1985 to 1st April 2026. Randomised controlled trials and observational studies with an unvaccinated comparison group were eligible if they assessed pre-exposure or post-exposure hepatitis A vaccination and reported effect estimates or sufficient data for their calculation. Estimates were pooled using a DerSimonian–Laird random-effects model. Risk of bias was assessed with RoB 2 and ROBINS-I, and certainty of evidence with GRADE. Ecological studies evaluating vaccine impact before and after implementation of paediatric vaccination programmes were analysed separately. The review was registered with PROSPERO (CRD420261308908).

**Findings:**

We included 21 publications in the meta-analysis and 19 studies in the population-level impact analysis. Pooled effectiveness of pre-exposure vaccination was 95·8% (95% CI 93·1–97·4; I^2^ = 29·9%), supported by moderate-certainty evidence. In paediatric populations, effectiveness was 97·5% (92·8–99·1; I^2^ = 20·1%) in randomised trials and 95·9% (93·0–97·6; I^2^ = 0·0%) in cohort studies. Effectiveness was 97·2% (93·8–98·8; I^2^ = 4·3%) for inactivated vaccines and 96·0% (93·2–97·6; I^2^ = 0·0%) for live attenuated vaccines. Pooled effectiveness of post-exposure vaccination was 96·9% (89·3–99·1; I^2^ = 89·7%), but certainty of evidence was very low. Ecological studies showed consistently high population-level impact, generally ranging from 80% to 100%.

**Interpretation:**

Hepatitis A vaccination provides very high protection in pre-exposure settings. Post-exposure vaccination may also be effective, but confidence in this estimate is limited by very low-certainty evidence and substantial heterogeneity. Population-level findings support the public health value of routine immunisation and suggest potential benefit in outbreak response.

**Funding:**

Cooperatio 31 fund, Health Sciences, Charles University, Prague, Czech Republic.

**Research in context**

**Evidence before this study**

We searched MEDLINE, Excerpta Medica Database (EMBASE), Biological Abstracts, and the Cochrane Central Register of Controlled Trials (CENTRAL), accessed through PubMed, Scopus, STNext, and the Cochrane Library, for studies published from 1 January 1985 to 1 April 2026, without language restrictions. The search used terms related to hepatitis A vaccination and vaccine effectiveness, including “immunization”, “HAV”, and “effectiveness”, together with their synonyms and database-specific controlled vocabulary. Previous reviews of hepatitis A vaccination have mainly focused on immunogenicity, antibody persistence, or programme experience rather than quantitatively synthesising vaccine effectiveness. No previous meta-analysis has jointly assessed pre-exposure and post-exposure vaccine effectiveness and linked these findings with evidence on population-level vaccine impact. Consequently, the extent to which vaccine effectiveness translates into population-level public-health benefit has remained incompletely defined.

**Added value of this study**

This study provides the first comprehensive quantitative synthesis of hepatitis A vaccine effectiveness in both pre-exposure and post-exposure use, together with evidence on population-level vaccine impact. Pre-exposure vaccination was associated with a pooled effectiveness of 95·8%, with certainty of evidence ranging from moderate overall to high in paediatric randomised trials. Although evidence for post-exposure vaccination was of very low certainty, the pooled protection estimate was similarly high at 96·9%. Ecological analyses showed consistently high population-level impact, generally ranging from 80% to 100%, with greater impact in settings with higher pre-vaccination incidence.

**Implications of all the available evidence**

Hepatitis A vaccination appears to provide very high protection and substantial public-health benefit. These findings support its use in routine immunisation programmes and outbreak response, particularly in settings with higher baseline incidence, while underscoring the need for more robust evidence on post-exposure use.

## Introduction

Hepatitis A virus (HAV) remains an important cause of acute viral hepatitis worldwide.^1^ Although infection is often asymptomatic in young children, older children, adolescents, and adults are more likely to develop clinically apparent and more severe disease, with rare progression to acute liver failure.^2^^,^^3^ In many settings, improvements in sanitation, hygiene, and access to clean water have reduced HAV transmission. However, this epidemiological transition has also increased the proportion of susceptible older individuals, shifting infection towards age groups in whom disease is more often symptomatic and severe.^4^

Against this background, hepatitis A vaccination has become an important public-health strategy, particularly in countries and regions from high to intermediate endemicity. In such settings, the World Health Organization recommends considering the introduction of hepatitis A vaccination into routine childhood immunisation programmes on the basis of local incidence and cost-effectiveness.^5^ More recently, a position paper from the European Society of Clinical Microbiology and Infectious Diseases Study Group for Viral Hepatitis advocated universal hepatitis A vaccination as a strategy to achieve global immunity and eventual eradication.^6^ However, direct estimates of clinical vaccine effectiveness remain important for interpreting the strength of this policy rationale across preventive settings. Universal childhood vaccination has been associated with substantial reductions in hepatitis A incidence and disease burden in several countries, supporting its potential value not only for individual protection but also for population-level prevention.

Both inactivated and live-attenuated HAV vaccines are available, although inactivated vaccines are the most widely used globally.^7^ These vaccines are administered mainly as monovalent formulations and are generally given in a two-dose schedule beginning at 12 months of age, although one-dose strategies have also been evaluated or implemented in some settings.^8^ HAV vaccines are safe and immunogenic, with long-term persistence of protective antibodies reported after two-dose schedules, and their effectiveness has been assessed in both pre-exposure and post-exposure settings.

Before HAV vaccines became available, post-exposure prevention relied mainly on immune globulin, which was effective but provided only short-term protection. Subsequent studies showed that HAV vaccine administered within 2 weeks of exposure was nearly as effective as immune globulin in preventing symptomatic hepatitis A, while also offering longer-term protection.^9^ Consequently, hepatitis A vaccination has also been incorporated into post-exposure prophylaxis strategies in many countries.

Despite the growing body of evidence, the overall public-health value of HAV vaccination across routine prevention and outbreak response has not been comprehensively quantified. In particular, findings from pre-exposure and post-exposure effectiveness studies have not been consistently synthesised together with evidence on population-level vaccine impact. This gap limits the ability of policy makers to understand how vaccine effectiveness translates into broader population-level public-health benefit. We therefore conducted HAVETO (Hepatitis A Vaccine Effectiveness: Trials and Observational Studies), a systematic review and meta-analysis, to quantify the effectiveness of HAV vaccination, assess the certainty of evidence, and summarise its population-level public-health impact across diverse populations and settings.

## Methods

This systematic review and meta-analysis was conducted in accordance with the Preferred Reporting Items for Systematic Reviews and Meta-Analyses (PRISMA) guidelines and the Meta-analysis Of Observational Studies in Epidemiology (MOOSE) guidelines.^10^, ^11^, ^12^ The protocol for the HAVETO meta-analysis was registered in the International Prospective Register of Systematic Reviews (PROSPERO; CRD420261308908).

### Literature search

We searched MEDLINE, EMBASE, Biological Abstracts, the Cochrane Central Register of Controlled Trials (CENTRAL), PubMed, and Scopus for eligible studies published between 1 January 1985 and 1 April 2026, without language restrictions. The search combined controlled vocabulary and free-text terms relating to hepatitis A or HAV, vaccination, immunisation, pre-exposure or post-exposure prophylaxis, and vaccine effectiveness, efficacy, immunogenicity, impact, outcomes, or reductions in disease occurrence. The search terms were adapted to the syntax and indexing system of each database. The complete database-specific search strategies are provided in the Supplementary Material (Supplementary Table S1). We also manually screened the reference lists of relevant review articles and meta-analyses to identify additional eligible studies.

### Study selection

Titles, abstracts, and potentially eligible full-text articles were screened independently by two reviewers (MP and IKL), with disagreements resolved by discussion until consensus was reached. For the primary analysis, we included randomised controlled trials and observational studies with unvaccinated comparison group if they assessed HAV vaccination used either for pre-exposure or post-exposure prophylaxis and reported effect estimates or sufficient data to calculate relative risks with corresponding confidence intervals (CIs). Eligible clinical studies were required to define hepatitis A outcomes using laboratory confirmation by IgM anti-HAV, either alone or in combination with additional clinical or laboratory criteria, such as compatible signs and symptoms, abnormal alanine aminotransferase levels, or HAV RNA detection. Studies relying solely on clinical symptoms or non-specific clinical hepatitis without IgM anti-HAV confirmation were not eligible for the primary analysis.

To complement the analysis of vaccine effectiveness, we also included ecological studies evaluating vaccine impact before and after implementation of HAV vaccination programmes in paediatric target populations. These studies were required to report an age-defined paediatric population, pre-vaccination and post-vaccination incidence rates, and vaccination coverage achieved in the target population.

Throughout this review, vaccine effectiveness refers to average effects estimated from randomised controlled trials and observational clinical studies with an unvaccinated comparison group, whereas vaccine impact refers to population-level changes in hepatitis A incidence estimated from ecological before-and-after studies of vaccination programmes.

### Data extraction and quality assessment

Extracted data included study characteristics, participant characteristics, interventions, comparators, and outcomes (PICO). Data were extracted by one reviewer (JM) and independently checked for accuracy by a second reviewer (MP). Any discrepancies were resolved by discussion until consensus was reached. Risk of bias in randomised trials was assessed using the revised Cochrane Risk of Bias tool (RoB 2), and observational studies were assessed using the Risk Of Bias In Non-randomized Studies of Interventions (ROBINS-I) tool.^13^^,^^14^ Risk-of-bias assessments were performed by one reviewer (MP) for all included studies and independently checked by a second reviewer (JM or JS), with disagreements resolved by discussion until consensus was reached.

The certainty of evidence for pooled vaccine-effectiveness estimates was assessed using the Grading of Recommendations Assessment, Development, and Evaluation (GRADE) approach.^15^ GRADE domain ratings were assigned according to predefined operational criteria applied to each pooled vaccine-effectiveness estimate. For study-level domains, including study limitations and indirectness, study-level classifications were summarised according to the meta-analytic contribution of individual studies, with a domain rated as serious when more than 50% of the weighted evidence indicated serious concerns. For estimate-level domains, including inconsistency and imprecision, ratings were based directly on the corresponding meta-analytic results.

The study limitations domain was derived from the overall risk-of-bias assessments of individual studies, dichotomised as non-serious limitations (low risk, some concerns, or moderate risk) or serious limitations (serious, high, or critical risk). Indirectness was judged according to whether the timing of vaccination matched the intended preventive setting: within 14 days of exposure for post-exposure vaccination and at least 4 weeks before follow-up for pre-exposure vaccination. Evidence meeting these criteria was rated as not serious for indirectness; evidence with an important mismatch was rated as serious. Inconsistency was assessed using the I^2^ statistic as a measure of between-study heterogeneity and was considered not serious when the summary value was below 50%. Imprecision was assessed according to the width of the 95% CI around the pooled vaccine-effectiveness estimate and was considered not serious when the interval width was less than 10 percentage points.

Publication bias was assessed using three criteria: differences between pooled estimates from fixed-effect and random-effects models, between the primary and trim-and-fill pooled estimates, and, when small-study effects were present, between the pooled estimate and the vaccine effectiveness extrapolated from the meta-regression model. This domain was judged as not serious only when all applicable criteria indicated differences of less than 10 percentage points (p.p.).

The starting certainty rating was high for randomised trials and low for observational studies. For pooled estimates based on both study designs or on observational studies alone, certainty of evidence was upgraded for large effect when pooled vaccine effectiveness was at least 90%, the lower bound of the prediction interval was at least 75%, and inconsistency and indirectness were not both rated as serious.

### Ecological studies

For ecological studies, extracted data included incidence rates before and after implementation of vaccination, corresponding observation periods, target age population, and estimated vaccination coverage. The vaccinated population was stratified into three age categories according to the upper age limit for which incidence rates were reported: younger than 5 years, 5–9 years, and 10–17 years. Vaccination coverage was classified according to the maximum reported estimate as low (≤60%), moderate (>60%–80%), or high (>80%). Pre-vaccination incidence was categorised as low (<10 cases), moderate (10–100 cases), or high (>100 cases per 100,000 person-years). Balance between the pre-vaccination and post-vaccination periods was assessed according to the difference in their duration, with a difference of at least 2 years classified as imbalance. Risk-of-bias assessment was not performed for ecological studies.

### Statistics

The primary meta-analysis estimated pooled overall vaccine effectiveness for pre-exposure and post-exposure HAV vaccination. Additional pooled estimates were generated according to vaccine type and study design for pre-exposure vaccination, and according to close-contact and outbreak settings for post-exposure vaccination. Vaccine effectiveness was derived from the effect size (ES) as (1 − ES) × 100%. Because between-study heterogeneity was anticipated, the primary quantitative synthesis used a random-effects model based on the DerSimonian-Laird method.

Additional analyses included pooled estimates from a fixed-effect model using the inverse-variance method, assessment of small-study effects by meta-regression with Egger's test, trim-and-fill adjustment for funnel-plot asymmetry, leave-one-out sensitivity analyses, and estimation of prediction intervals based on the standard error of between-study heterogeneity.^16^^,^^17^

A secondary analysis evaluated vaccine impact, derived from the incidence rate ratio (IRR) comparing hepatitis A incidence after and before implementation of vaccination, calculated as (1 − IRR) × 100%. The influence of selected study-level factors on vaccine impact was estimated using a Bayesian regression model with adaptive Metropolis–Hastings sampling. Regression coefficients were assigned weakly informative normal priors with mean 0 and standard deviation 10. Posterior summaries were expressed as differences in vaccine impact with corresponding 95% highest posterior density credible intervals (95% CrI). Associations were considered credible when the 95% CrI excluded 0. Independent variables were age of the target population, pre-vaccination incidence, balance between the pre-vaccination and post-vaccination periods, and estimated vaccination coverage.

Statistical analyses were performed using Stata version 18·0 (StataCorp, College Station, TX, USA), with statistical significance set at α = 0·05 and all estimates reported with two-sided 95% confidence intervals (95% CIs).

### Role of the funding source

This work was supported by the Cooperatio 31 fund, Health Sciences, Charles University, Prague, Czech Republic. The fund had no role in the study design, data analysis, data interpretation, or writing of this report.

## Results

### Study characteristics

**Fig. 1:**
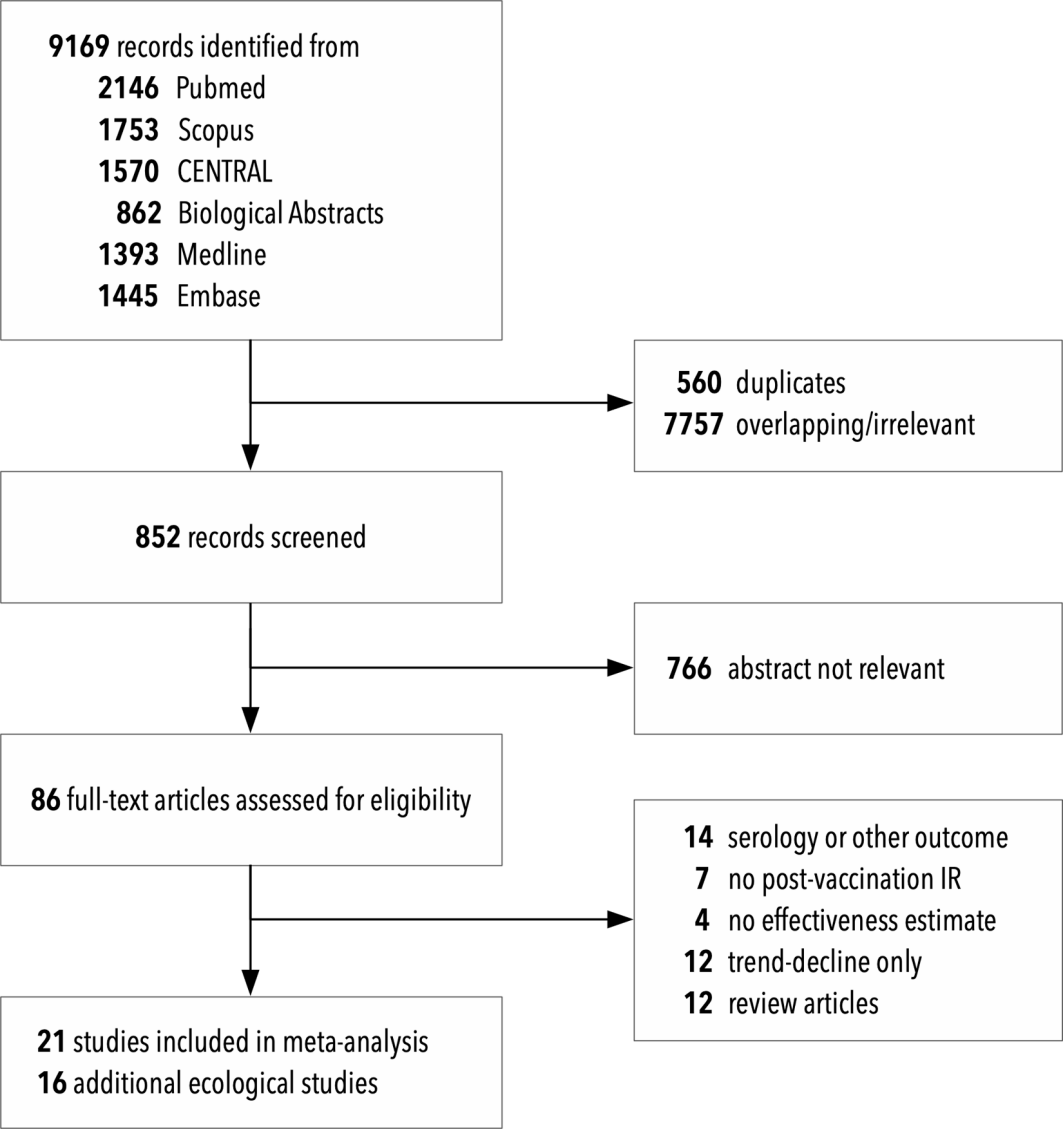
Flow diagram of study selection.

A total of 21 publications were eligible for inclusion in the meta-analysis, of which three also assessed the impact of HAV vaccination in paediatric populations (Fig. 1). Only randomised controlled trials (RCTs) and cohort studies published between 1990 and 2017 were identified. Full domain-level risk-of-bias assessments are provided separately for RCTs assessed using RoB 2 and for cohort studies assessed using ROBINS-I in Supplementary Figures S1 and S2, respectively. The pre-exposure vaccine-effectiveness meta-analysis included 15 estimates derived from six RCTs and eight cohort studies, whereas post-exposure vaccine effectiveness was evaluated using eight estimates from one RCT and seven cohort studies (Table 1).

**Table 1:** Characteristics of studies included in the meta-analysis of hepatitis A vaccine effectiveness.

Author, year	Country	Design	Study period	Vaccination	Population (age)	Vaccine (doses)	Unvaccinated n/events	Vaccinated n/events	Risk window^b^	Follow-up	VE, %^a^	Risk of bias	Comments
Riedemann, 1992^18^	Chile	RCT	1990–1992	Pre-exposure	Children (6–15)	Inactivated (≥1)	130/1	127/0	1 month	12 months	100^a^	Some concerns	Excluded 3 cases <1 month; Havrix
Werzberger, 1992^19^	USA	RCT	1991–1991	Pre-exposure	Children (2–16)	Inactivated (1)	496/25	498/0	50 days	<5 months	100	Some concerns	Vaqta
Innis, 1994^20^	Thailand	RCT	1991–1993	Pre-exposure	Children (1–16)	Inactivated (≥2)	19,120/38	19,037/2	4·6 months	<18 months	95	Some concerns	0–1–6-schedule; Havrix
Richtmann, 1996^21^	Brazil	RCT	1994–1995	Pre-exposure	Children (0·6–4·8)	Inactivated (3)	27/5	35/0	2 months	12 months	100	High risk	1·0 mL and 0·5 mL doses; 0–1–6-schedule; Avaxim
Gong, 2003^22^	China	RCT	1998–2002	Pre-exposure	Children (1·5–10)	Live (1)	112,250/71	100,735/2	1 month	36 months	97	Some concerns	Estimated follow-up; LA-1 live vaccine
Mayorga Pérez, 2003^23^	Nicaragua	RCT	2000–2002	Pre-exposure	Children (1·5–6)	Inactivated (1)	112/17	118/0	42 days	15 months	100	Some concerns	Estimated study period; Epaxal
Mao, 1997^24^	China	Cohort	1989–1993	Pre-exposure	Children (1–15)	Live (1)	242,168/495	18,102/0	NR	48 months	100	Serious	Person-years; H2 live vaccine
Zhao, 2000^25^	China	Cohort	1996–1998	Pre-exposure	Children (3–11)	Live (1)	422/21	356/1	12 months	<36 months	95	Serious	Estimated study period/follow-up; H2 live vaccine
Averhoff, 2001^26^	USA	Cohort	1995–2000	Pre-exposure	Children (2–17)	Inactivated (≥1)	15,193/26	29,789/1	NR	60 months	98	Moderate	Vaccinated case dosed 3 days before onset; Vaqta
Xu ZY, 2002^27^	China	Cohort	1996–2000	Pre-exposure	Children (1–12)	Live (1)	31,224/22; 55,633/38	27,668/1; 58,955/2	NR	<36 months	95	Moderate	Estimated study period; same VE for both vaccines; H2 or LA-1 live vaccine
Fisenka, 2008^28^	Belarus	Cohort	2003–2006	Pre-exposure	Children (1–17)	Inactivated (2)	210,900/131	65,171/2	NR	48 months	95	Moderate	0–6-schedule; 2 cases within 14 days; mostly Avaxim
Mayorga, 2016^29^	Nicaragua	Cohort	2005–2012	Pre-exposure	Children (2–17)	Inactivated (1)	96/58	96/1	12 months	90 months	98	Critical	Epaxal
Lin, 2018^30^	Taiwan	Cohort	2015–2017	Pre-exposure	HIV–positive adults (≥19; median 34)	Inactivated (≥1)	532/60	1001/5	Median 3 months	48 months	96	Moderate	All vaccinated cases occurred at a median of 3 months post-vaccination; Havrix or Vaqta
Im, 2020^31^	South Korea	Cohort	2013–2016	Pre-exposure	Military personnel (18–31)	Inactivated (1)	1,020,450/21	603,550/3	7 days	<24 months	76	Moderate	Person-years; Havrix
Príkazský, 1994^32^	Slovakia	Cohort	1991–1993	Post-exposure	Outbreak–area schoolchildren (6–15)	Inactivated (≥1)	157/8	404/1	NR	90 days	95^a^	Serious	Havrix
McMahon, 1996^33^	USA	Cohort	1994–1995	Post-exposure	Outbreak community (<34)	Inactivated (1)	997/120	1829/38	NR	420 days	83^a^	Serious	Likely vaccinated >2 weeks after exposure; Havrix
Sagliocca, 1999^34^	Italy	RCT	1997	Post-exposure	Household contacts (1–40)	Inactivated (1)	75/10	71/2	14 days	45 days	79	Some concerns	Havrix
Selnikova, 2008^35^	Ukraine	Cohort	2003	Post-exposure	Outbreak–area children (2–14)	Inactivated (1)	1496/68	908/1	NR	<77 days	98	Serious	Vaccinated case at 7 days; Avaxim
Shen, 2008^36^	China	Cohort	2006–2007	Post-exposure	Outbreak–area students (5–19); Close contacts (<49)	Inactivated (1)	2571/4; 115/4	3364/0; 108/0	NR; <31 days	120 days; 60 days	100	Serious	Estimated follow-up; same VE for both populations; predefined 15-day post-vaccination window; Healive
Freeman, 2014^37^	Australia	Cohort	2008–2010	Post-exposure	Close contacts (1–85)	Inactivated (1)	57/9	144/1	14 days	<50 days	96	Serious	Brand unspecified
Parrón, 2017^38^	Spain	Cohort	2006–2012	Post-exposure	Close contacts (<45)	Inactivated (1)	611/184	2381/17	14 days	50 days	98	Serious	Brand unspecified

RCT = randomised controlled trial. VE = vaccine effectiveness. NR = not reported.

a VE values were calculated from raw event counts.

b Risk window was defined as the interval between vaccination and the start of follow-up for HAV infection in pre-exposure studies, and as the interval between HAV exposure and vaccination in post-exposure studies. Risk of bias was assessed using RoB 2 for randomised trials and ROBINS-I for cohort studies.

Pre-exposure vaccination was assessed almost exclusively in individuals younger than 18 years, with only two cohort studies conducted in adults. By contrast, post-exposure vaccination was evaluated across all age groups, and only three estimates were based on studies in children or adolescents younger than 19 years. Most studies assessed inactivated HAV vaccines, generally after administration of at least one dose. Only four studies evaluated one-dose pre-exposure vaccination with live attenuated HAV vaccines, all in Chinese children younger than 15 years.

All RCTs specified the risk window, defined either as at least 1 month between vaccination and the start of follow-up for HAV infection or, in post-exposure studies, as no more than 14 days between potential HAV exposure and vaccination. By contrast, cohort studies of pre-exposure vaccination either did not report the risk window or relied on ad hoc estimation based on the timing of HAV case occurrence. In post-exposure studies, the risk window was usually defined as within 14 days in close-contact settings, whereas it was not specified in outbreak-area studies. Among studies evaluating pre-exposure vaccination, follow-up ranged from at least 5 months to 7·5 years; in post-exposure studies, the shortest follow-up was 45 days.

### Effectiveness of pre-exposure vaccination

**Fig. 2:**
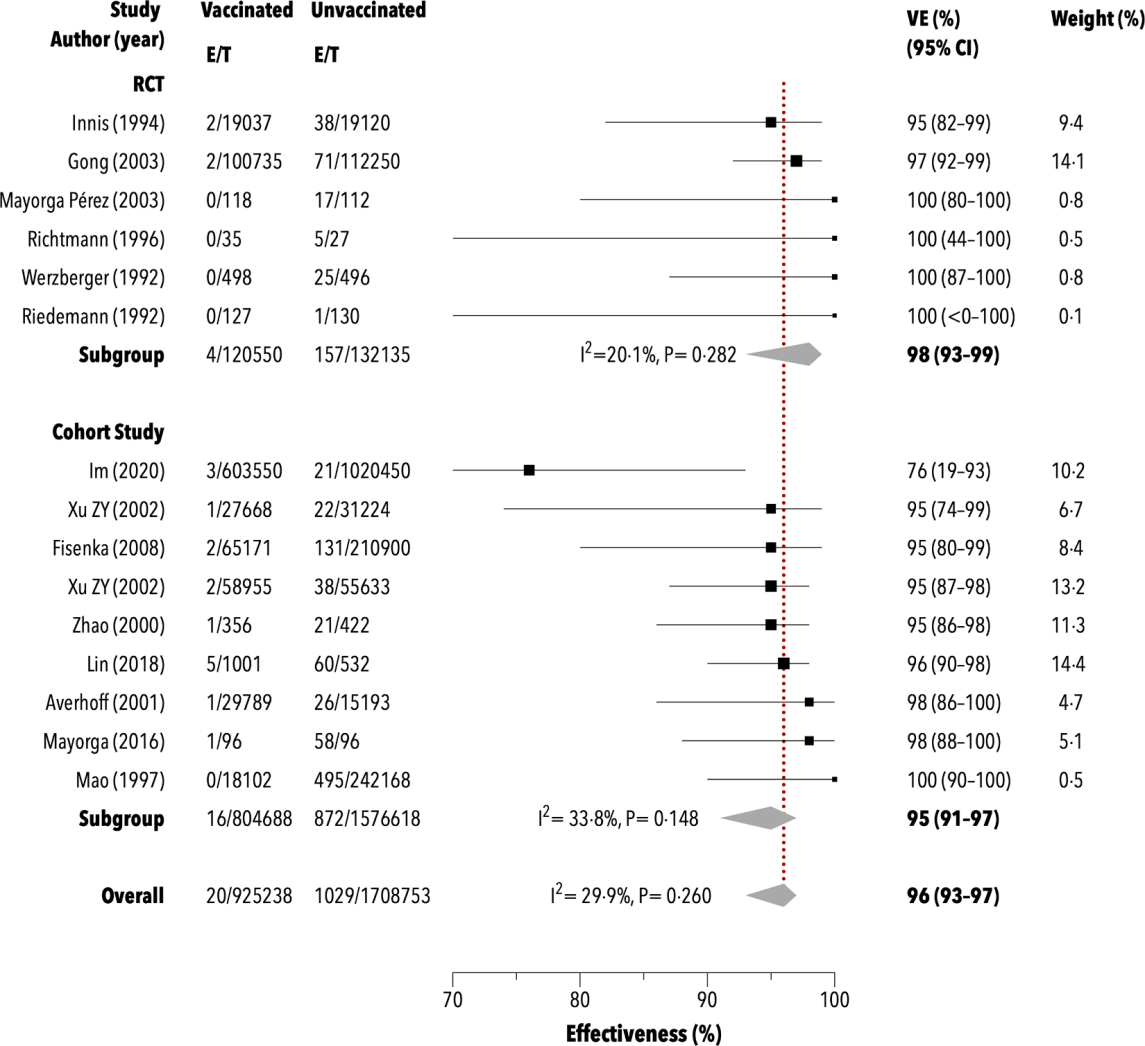
Forest plot of effectiveness estimates for pre-exposure hepatitis A vaccination by study design. Squares represent study-specific vaccine effectiveness (VE) estimates, with size proportional to study weight, and horizontal lines indicate 95% CIs. Diamonds represent pooled subgroup and overall estimates from the random-effects meta-analysis. E/T indicates number of HAV events/total participants. VE = vaccine effectiveness. RCT = randomised controlled trial.

The pooled effectiveness of pre-exposure HAV vaccination was 95·8% (95% CI 93·1–97·4%; I^2^ = 29·9%), irrespective of study design. This finding was also supported by the observed event counts, with 20 HAV infections among 925,238 recipients of at least one vaccine dose compared with 1029 infections among 1,708,753 unvaccinated individuals (Fig. 2). Leave-one-out sensitivity analyses showed that the pooled estimate for pre-exposure HAV vaccination was robust to the exclusion of individual articles (Supplementary Table S2).

The evidence base consisted predominantly of studies in children and adolescents younger than 18 years, with only two cohort studies conducted in adults. Although small-study effects were detected and the funnel plot suggested asymmetry driven mainly by smaller studies with larger vaccine effects, publication bias was judged as not serious because the pooled estimates from the fixed-effect model, trim-and-fill analysis, and meta-regression extrapolation each differed from the primary estimate by less than 10 p.p (Table 2, Supplementary Table S3, Supplementary Figure S3). The primary pooled estimate was not affected by serious limitations, inconsistency, indirectness, or imprecision. Because this result was derived from both RCTs and cohort studies and was supported by a prediction interval of 86·0–98·7%, the certainty of evidence was upgraded from low to moderate for large effect.

**Table 2:** Pooled effectiveness of hepatitis A vaccination according to indication, vaccine type, study design, and exposure setting.

Vaccination	Subgroup	Age group	Estimates, n	Pooled VE (95% CI)	I^2^, %	Imputed VE (95% CI)	Prediction interval, %	Certainty of evidence
Pre-exposure	Overall	Any	15	95·8 (93·1–97·4)	29·9	95·2 (91·6–97·3)	86·0–98·7	Moderate
	Inactivated vaccine	<18 years	8	97·2 (93·8–98·8)	4·3	96·6 (90·9–98·7)	90·9–99·1	Moderate
	Live vaccine	<18 years	5	96·0 (93·2–97·6)	0·0	95·7 (91·8–97·8)	90·5–98·3	Moderate
	RCTs	<18 years	6	97·5 (92·8–99·1)	20·1	96·8 (89·3–99·1)	77·4–99·7	High
	Cohort studies	<18 years	7	95·9 (93·0–97·6)	0·0	95·8 (92·5–97·7)	91·7–98·0	Low
Post-exposure	Overall	Any	8	96·9 (89·3–99·1)	89·7	96·9 (89·3–99·1)	<0–99·9	Very low
	Close-contact setting	Any	4	95·3 (79·4–98·9)	67·0	94·4 (73·9–98·8)	<0–100·0	Very low
	Outbreak-area setting	Any	4	98·4 (79·3–99·9)	89·7	98·4 (79·3–99·9)	<0–100·0	Very low

Imputed VE was estimated using the trim-and-fill method. Prediction intervals with lower bounds below 0 are presented as <0 for readability. Certainty of evidence was assessed using the GRADE approach. VE = vaccine effectiveness. RCT = randomised controlled trial.

In an additional sensitivity analysis restricted to 11 pre-exposure studies without high overall risk of bias according to RoB 2 or serious/critical overall risk of bias according to ROBINS-I, pooled vaccine effectiveness remained similar to the primary estimate at 95·3% (95% CI 92·0–97·2%; I^2^ = 30·4%). The certainty of evidence for this restricted analysis nevertheless remained low, because exclusion of studies with higher risk of bias did not resolve the remaining concerns, particularly indirectness.

When restricted to paediatric populations, pooled vaccine effectiveness was 97·5% (95% CI 92·8–99·1%; I^2^ = 20·1%) in RCTs and 95·9% (95% CI 93·0–97·6%; I^2^ = 0·0%) in cohort studies (Table 2). The RCT-based estimate was supported by high-certainty evidence and a prediction interval of 77·0–100·0%. The cohort-based estimate, with a prediction interval of 91·7–98·0%, met the criterion for no serious concern in all domains except indirectness, which was rated as serious because the risk window was frequently not specified; the overall certainty of evidence therefore remained low.

Among paediatric populations, pooled effectiveness was 97·2% (95% CI 93·8–98·8%; I^2^ = 4·3%) for inactivated vaccines and 96·0% (95% CI 93·2–97·6%; I^2^ = 0·0%) for live attenuated vaccines. Both estimates were supported by moderate-certainty evidence, and both prediction intervals had lower bounds above 90%.

### Effectiveness of post-exposure vaccination

**Fig. 3:**
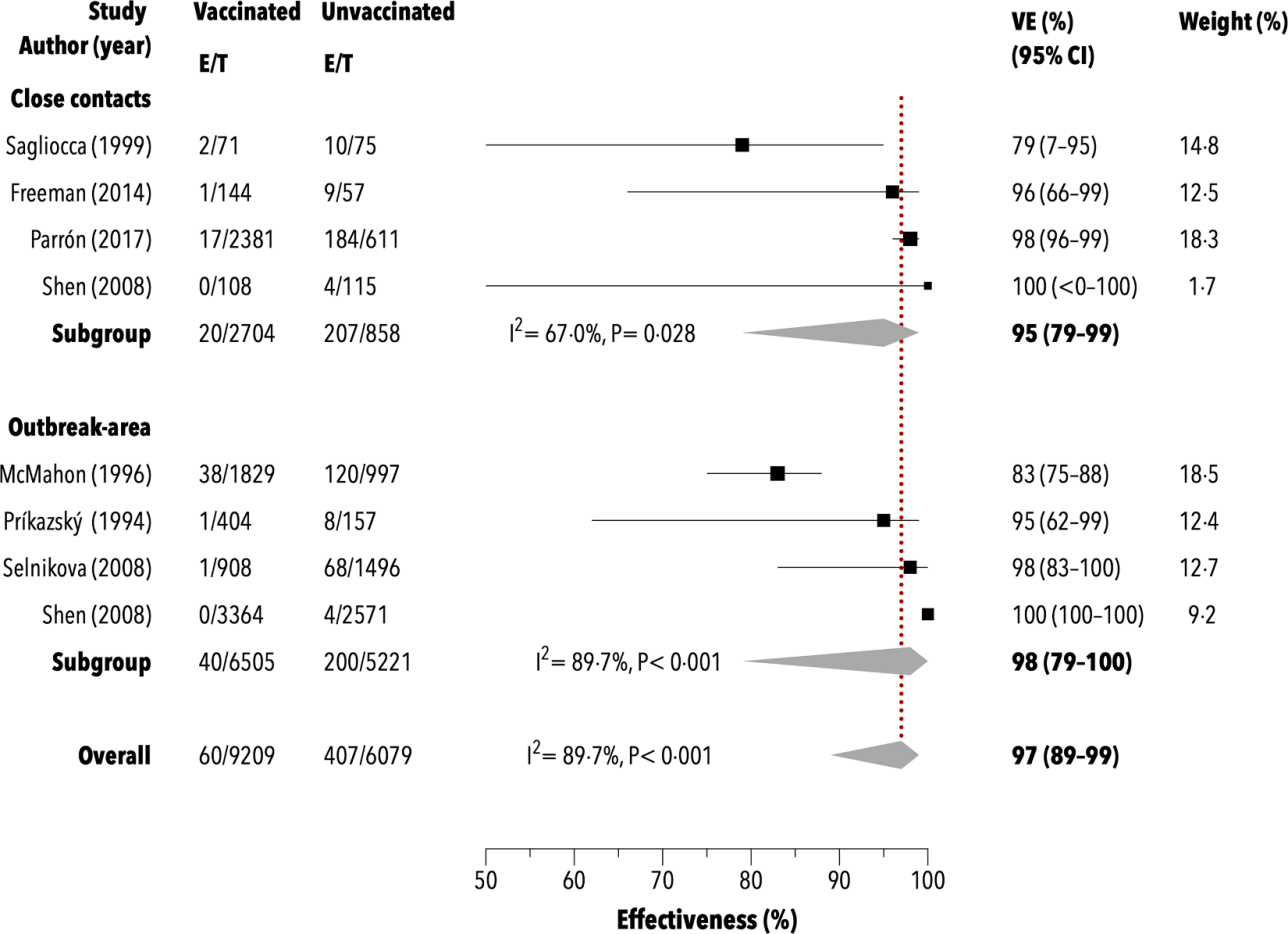
Forest plot of effectiveness estimates for post-exposure hepatitis A vaccination by exposure setting. Squares represent study-specific vaccine effectiveness (VE) estimates, with size proportional to study weight, and horizontal lines indicate 95% CIs. Diamonds represent pooled subgroup and overall estimates from the random-effects meta-analysis. E/T indicates number of HAV events/total participants. VE = vaccine effectiveness.

The pooled effectiveness of post-exposure vaccination, based on eight estimates, was 96·9% (95% CI 89·3–99·1%; I^2^ = 89·7%). Estimates were similar in studies of close contacts (95·3%, 95% CI 79·4–98·9%; I^2^ = 67·0%) and outbreak-area populations (98·4%, 79·3–99·9%; I^2^ = 89·7%). Overall, seven eligible studies documented HAV infection in 60 of 9209 post-exposure vaccinated individuals compared with 407 of 6079 unvaccinated individuals (Fig. 3). Leave-one-out sensitivity analyses showed that sequential exclusion of individual articles did not materially alter the pooled estimate for post-exposure HAV vaccination, indicating that the overall direction and magnitude of the effect were not driven by any single study (Supplementary Table S2).

Despite these high pooled estimates, the certainty of evidence was very low, mainly because of serious limitations, inconsistency, and indirectness across studies (Table 2, Supplementary Table S2, Supplementary Figure S4). This uncertainty was further reflected by a prediction interval with a negative lower bound.

### Population-level impact of vaccination programmes

The population-level vaccine impact was assessed using 19 ecological studies conducted between 1997 and 2024, three of which also had a cohort design (Supplementary Table S4). Because this analysis focused on vaccine impact in vaccinated target populations, it included only children and adolescents younger than 17 years in universal or routine vaccination programmes. Accordingly, the studies were conducted in countries or regions where HAV vaccination had been incorporated into national or local immunisation programmes. China was the only country in which both inactivated and live attenuated HAV vaccines were used.

In the Bayesian regression model, the estimated vaccine impact was 83·9% (95% CrI 79·3–88·5%) for vaccinated children younger than 5 years with vaccination coverage up to 60% and a pre-vaccination incidence rate below 10 cases per 100,000 person-years. Compared with this reference category, vaccine impact was estimated to be 8·2 p.p. higher (95% CrI 2·1–14·2 p.p.) in settings with pre-vaccination incidence rates above 100 cases per 100,000 person-years. Differences according to age strata, pre/post-period balance, and vaccination coverage were not clearly supported (Supplementary Figure S5).

**Fig. 4:**
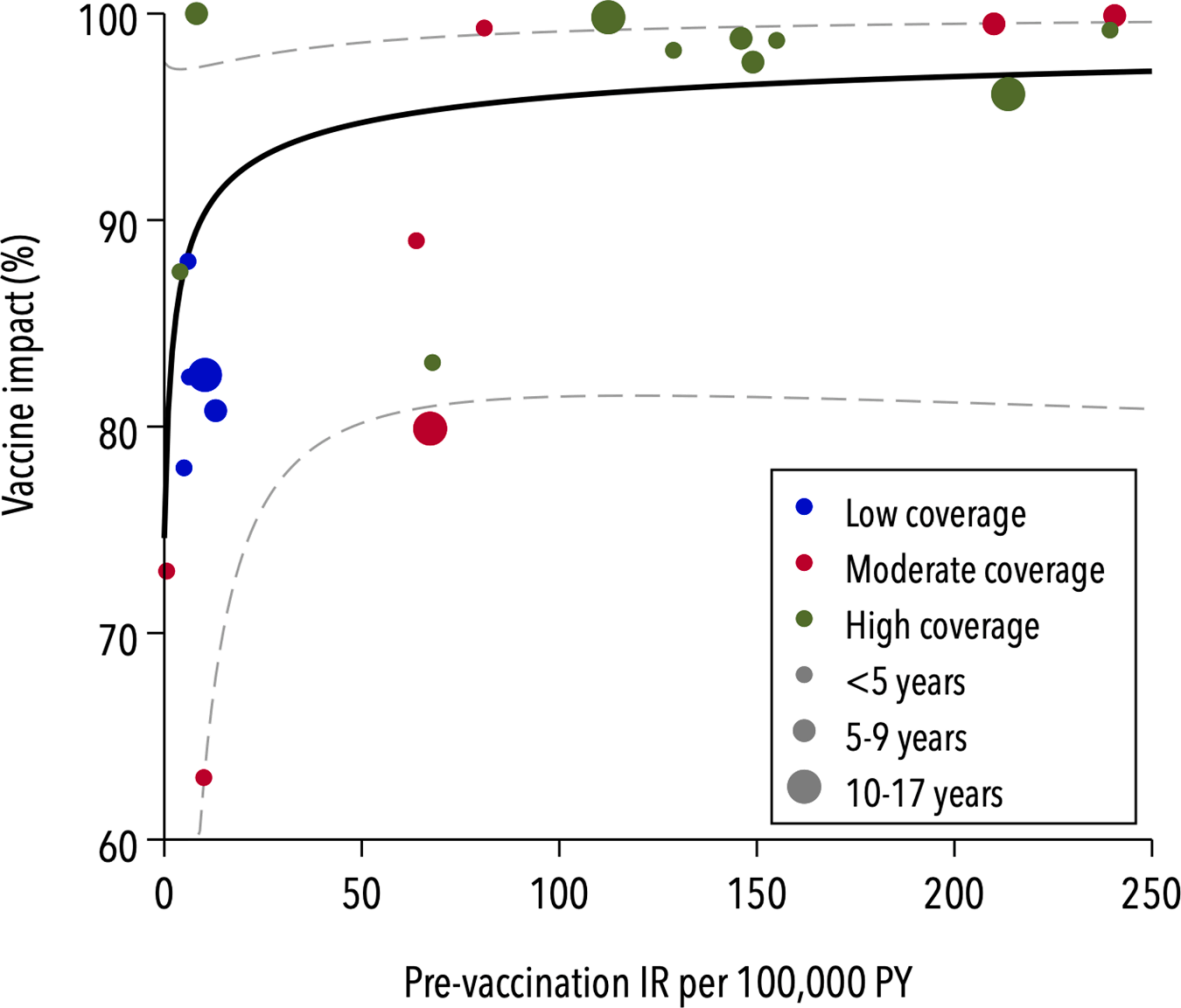
Relationship between pre-vaccination incidence and hepatitis A vaccine impact in paediatric target populations. Each circle represents one study estimate. Circle colour indicates vaccination coverage (low, moderate, or high), and circle size indicates the age group targeted for vaccination (<5 years, 5–9 years, or 10–17 years). The solid line shows the fitted dependence of vaccine impact on pre-vaccination incidence rate (IR) from the Bayesian regression model, and dashed lines indicate 95% credible intervals. IR = incidence rate. PY = person-years.

Overall, population-level vaccine impact was high, generally ranging from about 80% to 100%, with a non-linear increase at higher pre-vaccination incidence rates and a plateau at very high levels (Fig. 4).

## Discussion

This systematic review and meta-analysis show that hepatitis A vaccination provides very high protection at the individual level and substantial benefit at population level. Pre-exposure vaccination was associated with a pooled effectiveness of 96%, supported overall by moderate-certainty evidence and by high-certainty evidence from paediatric randomised controlled trials. Post-exposure vaccination was also associated with a high pooled effectiveness of 97%, although the certainty of evidence was very low. Ecological studies further showed consistently high vaccine impact in paediatric target populations, with estimates generally ranging from 80% to 100%. Taken together, these findings support the public-health value of hepatitis A vaccination for both routine prevention and outbreak response.

Evidence on pre-exposure vaccination was derived predominantly from studies in children and adolescents, with only two cohort studies conducted in adults, specifically among military personnel and people living with HIV.^30^^,^^31^ Despite the scarcity of data in adults, there was no clear indication that effectiveness differed substantially from that observed in paediatric populations, with estimates of 96% in people living with HIV and 76% in healthy military personnel. The evidence base therefore supports strong protection in children and adolescents, whereas the evidence in adults remains more limited.

In analyses restricted to paediatric populations, pooled effectiveness remained high in both randomised trials and cohort studies. The RCT-based estimate reached 98% and was supported by high-certainty evidence, whereas the cohort-based estimate remained similarly high at 96% but was supported by low-certainty evidence because of serious indirectness, mainly related to incomplete reporting of the risk window used to define vaccine-protected follow-up. Nevertheless, the consistency of the pooled estimates and the narrow prediction interval in cohort studies support the robustness of the protective effect observed for pre-exposure vaccination.

No substantial difference was observed between inactivated and live attenuated vaccines in paediatric populations. Pooled effectiveness was 97% for inactivated vaccines and 96% for live attenuated vaccines, with moderate-certainty evidence for both. These findings suggest that the very high protection associated with hepatitis A vaccination is not limited to a single vaccine platform, although the evidence for live attenuated vaccines came exclusively from studies in Chinese children receiving a single dose.

The pooled effectiveness of post-exposure vaccination was similarly high and showed no major difference between close-contact settings and outbreak-area use. This finding supports the role of hepatitis A vaccination not only in routine prevention but also in outbreak-response strategies. However, the evidence base for post-exposure vaccination was derived almost entirely from observational studies, with only one randomised trial.^34^ These studies were affected by serious limitations, inconsistency, and indirectness, resulting in very low certainty of evidence and an uncertain prediction interval. Therefore, although the magnitude of protection was consistently high, conclusions regarding post-exposure use should remain more cautious than those for pre-exposure vaccination. In addition, although outside the scope of this meta-analysis, one small molecular study has suggested the possibility that vaccination during recent or ongoing HAV exposure could contribute to selective pressure on antigenic variants, further supporting a cautious interpretation of post-exposure vaccination findings.^39^

Even so, the convergence of findings across exposure settings is notable. The pooled estimate for post-exposure vaccination was similar to that observed for pre-exposure vaccination, and this pattern was consistent across both close-contact and outbreak-area studies. For public-health decision making, this finding is relevant because it suggests that hepatitis A vaccination can contribute meaningfully to outbreak control while also providing longer-term protection than passive immunisation alone.^9^

The ecological analyses complement the vaccine-effectiveness findings by showing that vaccination programmes were associated with substantial reductions in hepatitis A incidence in paediatric target populations. Vaccine impact was higher in settings with greater pre-vaccination incidence, increasing by about 8 percentage points when pre-vaccination incidence exceeded 100 cases per 100,000 person-years. By contrast, no clear association was observed for age of the target population, vaccination coverage, or balance between the pre-vaccination and post-vaccination periods. These findings suggest that baseline epidemiological context may be a more important determinant of programme impact than differences in programme design captured by the available data.

This pattern is particularly relevant for countries and regions undergoing epidemiological transition from high to intermediate endemicity. In such settings, the accumulation of susceptible older children, adolescents, and adults can increase the burden of symptomatic disease, strengthening the rationale for vaccination as a public-health intervention.^5^ The consistently high population-level impact observed across ecological studies, often approaching or exceeding 90% in high-incidence settings, indicates that routine childhood vaccination can produce major reductions in disease burden where transmission has remained substantial before programme introduction.

Although estimated vaccination coverage in target populations ranged from 40% to 95%, this variability did not clearly explain differences in vaccine impact. One possible explanation is that vaccination of younger children reduces transmission more broadly, including in older age groups that were not themselves targeted for vaccination. This interpretation is supported by previous studies showing declines in hepatitis A incidence among unvaccinated adults after introduction of childhood vaccination programmes.^8^^,^^40^, ^41^, ^42^, ^43^ The population-level findings of the present study are consistent with that interpretation and support the view that hepatitis A vaccination can generate benefits beyond directly vaccinated groups.

To our knowledge, this is the first meta-analysis to jointly evaluate pre-exposure and post-exposure hepatitis A vaccination and to integrate these findings with evidence on population-level vaccine impact. Previous evidence syntheses have mainly focused on immunogenicity in specific populations, long-term antibody persistence, or programme descriptions, or they have been limited to systematic reviews without quantitative synthesis.^44^, ^45^, ^46^, ^47^, ^48^ By combining vaccine-effectiveness estimates from clinical studies with ecological evidence on vaccine impact, our analysis provides a more policy-relevant synthesis of the public-health value of hepatitis A vaccination.^49^^,^^50^

A major strength of this study is the integration of evidence across different indications, study designs, and levels of inference. Pre-exposure vaccination was supported by high-certainty evidence from paediatric trials and by consistent findings from observational studies. Post-exposure vaccination showed similarly high pooled protection, albeit with much lower certainty. In addition, the ecological analysis made it possible to assess whether high vaccine effectiveness translated into measurable population-level reductions in disease, and to examine factors associated with variation in impact across settings.

This study also has limitations. First, the available evidence was methodologically heterogeneous, particularly with respect to risk-window definition, follow-up duration, and study design. Second, pooled estimates for pre-exposure vaccination referred to vaccination with at least one dose, because the included studies often did not permit dose-specific analysis. As a result, the extent to which two-dose schedules confer additional effectiveness beyond one dose could not be reliably assessed. This question is particularly relevant because one study of single-dose vaccination in military personnel reported lower effectiveness than most other studies.^31^ Third, evidence in adults was limited, especially for routine pre-exposure use. Fourth, vaccine-specific comparisons were constrained by the small number of estimates available for individual products. Finally, follow-up in most studies was limited to a few months or years, which precluded robust assessment of long-term persistence of protection, although one study reported effectiveness of 98% over 7·5 years.^29^

In conclusion, hepatitis A vaccination appears to provide very high protection at individual level and substantial benefit at population level. The evidence is strongest for pre-exposure vaccination in paediatric populations, while available data also suggest an important role for vaccination in outbreak response, although the certainty of evidence for post-exposure use remains lower. These findings strengthen the public-health case for hepatitis A vaccination in routine childhood immunisation programmes and support its potential value in outbreak-control strategies, particularly in settings with higher baseline incidence, while underscoring the need for more robust evidence on post-exposure use and on effectiveness in adult populations.

## Contributors

MP, JM, JS, PD, and JR conceptualized and designed the study. MP, JS, and IKL curated the data. MP, IKL, JS, and JM accessed and verified the underlying study data. JM and MP contributed to data interpretation. MP did the formal data analysis. MP, JM, PD, JS, and IKL contributed to the methodology. PD ensured funding acquisition. MP and JR administered the project. MP supervised the project. MP, JM and IKL wrote the original draft, and all authors contributed to data interpretation and writing, reviewing, and editing this manuscript. All authors read and approved the final version of the manuscript.

## Data sharing statement

The data that support the findings of this study are available from the corresponding author upon reasonable request.

## Declaration of interests

We declare no competing interests.
