# Supplementary material for: Hepatitis A vaccination and its public-health impact in routine prevention and outbreak control: a systematic review and meta-analysis
Source: eClinicalMedicine. 2026 Aug 11;99:104144. doi: 10.1016/j.eclinm.2026.104144 (PMC13486769; doi:10.1016/j.eclinm.2026.104144)
Supplement: Supplementary file 1 [file mmc1.pdf]

Hepatitis A vaccination and its public-health impact in routine prevention and outbreak control: a systematic review and meta-analysis

Supplementary Material.

Table of content

S-Table 1. Search strategy

S-Table 2. Leave-one-out sensitivity analysis of pooled vaccine effectiveness estimates for pre- and post-exposure hepatitis A vaccination

S-Table 3. Assessment of certainty of evidence for pooled hepatitis A vaccine effectiveness estimates

S-Table 4. Ecological studies evaluating the impact of hepatitis A vaccination on incidence in target populations

S-Figure 1. Summary of RoB 2 judgments across included randomised controlled trials

S-Figure 2. Summary of ROBINS-I judgments across included cohort studies

S-Figure 3. Funnel plot for pre-exposure hepatitis A vaccination.

S-Figure 4. Funnel plot for post-exposure hepatitis A vaccination.

S-Figure 5. Factors associated with differences in hepatitis A vaccine impact in paediatric target populations

S-Table 1. Search strategy

Criteria

PubMed Database

("Hepatitis A Vaccines"[Mesh] OR "Hepatitis A/prevention and control"[Mesh] OR "Hepatitis A/immunology"[Mesh] OR "Hepatitis A Virus, Human/immunology"[Mesh] OR "Hepatitis A virus/immunology"[Mesh] OR (("Hepatitis A"[Mesh] OR "Hepatitis A virus"[Mesh] OR "Hepatitis A Virus, Human"[Mesh] OR "Hepatitis A"[Title/Abstract] OR "type A Hepatitis"[Title/Abstract] OR "Hepatitis type A"[Title/Abstract] OR HAV[Title/Abstract]) AND ("Vaccines"[Mesh] OR "Vaccination"[Mesh] OR "Immunization"[Mesh] OR "Post-Exposure Prophylaxis"[Mesh] OR "Pre-Exposure Prophylaxis"[Mesh] OR "Immunization Programs"[Mesh] OR "Primary Prevention"[Mesh] OR vaccin*[Title/Abstract] OR immunis*[Title/Abstract] OR immuniz*[Title/Abstract] OR prophyla*[Title/Abstract] OR Immunoprophylaxis OR "Immuno prophylaxis"[Title/Abstract: ~3])) OR (("Viral Hepatitis Vaccines"[Mesh] OR "Hepatitis, Viral, Human/prevention and control"[Mesh] OR "Hepatitis, Viral, Human/immunology"[Mesh] OR "Hepatitis/prevention and control"[Mesh] OR "Hepatitis/immunology"[Mesh] OR "Hepatovirus/immunology"[Mesh]) AND ("Hepatitis A"[Mesh] OR "Hepatitis A virus"[Mesh] OR "Hepatitis A Virus, Human"[Mesh] OR "Hepatitis A" OR "type A Hepatitis" OR "Hepatitis type A" OR HAV[Title/Abstract]))) AND ("Vaccine Efficacy"[Mesh] OR effectiveness[Title/Abstract] OR effect*[Title] OR efficacy[Title/Abstract] OR immunogen*[Title/Abstract] OR impact*[Title/Abstract] OR reduction[Title/Abstract] OR reduc*[Title] OR durability[Title/Abstract] OR duration[Title/Abstract] OR "protective effect"[Title/Abstract] OR "real-world"[Title/Abstract] OR "real world"[Title/Abstract] OR "Treatment Outcome"[Title/Abstract] OR "Outcome"[Title] OR "Treatment Outcome"[Mesh])

Scopus Database

(TITLE (impact* OR reduc* OR effect* OR outcome) OR TITLE-ABS-KEY (effectiveness OR efficacy OR immunogen* OR reduction OR durability OR duration OR "protective effect" OR "real-world" OR "real world" OR "Treatment Outcome")) AND TITLE-ABS-KEY (vaccin* OR immunis* OR immuniz* OR prophyla* OR Immunoprophylaxis OR "Immuno prophylaxis") AND TITLE-ABS-KEY ("Hepatitis A" OR "type A Hepatitis" OR "Hepatitis type A") AND KEY ("Hepatitis A Vaccine")

EBM Reviews - Cochrane Central Register of Controlled Trials <January 2026>

1 (effectiveness or efficacy or immunogen* or impact* or reduction or durability or duration or "protective effect" or "real-world" or "real world").ti,ab. 1005444

2 (reduc* or effect*).ti. 590186

3 (vaccin* or immunis* or immuniz* or prophyla* or Immunoprophylaxis or "Immuno prophylaxis").ti. 45923

4 ("Hepatitis A" or "type A Hepatitis" or "Hepatitis type A").ti,ab. 1012

5 Hepatitis A Vaccines.sh,kw. 285

6 Viral Hepatitis Vaccines.sh,hw,kw,fx,xm,xs. 303

7 ("Hepatitis A" or "Hepatitis A virus" or "Hepatitis A Virus, Human").sh,hw,kw,fx,xm,xs. 1898

8 (Vaccines or Vaccination or Immunization or "Post-Exposure Prophylaxis" or "Pre-Exposure Prophylaxis" or "Immunization Programs" or "Primary Prevention").sh,hw,kw,fx,xm,xs.26838

9 Vaccine Efficacy.sh,hw,kw,fx,xm,xs. 173

10 1 or 2 or 9 1343574

11 3 or 8 54047

12 1 or 2 1343566

13 9 or 11 or 12 1363741

14 4 or 7 2434

15 Hepatitis.ti,sh,hw,kw,ab,fx,xm,xs. 23752

16 11 and 15 3717

17 13 and 14 1823

18 5 or 17 1823

19 13 and 15 15113

20 6 or 19 15113

21 limit 18 to yr="1898 - 2011" 990 - display all

22 limit 18 to yr="2012 -Current" 833 - display 1-600

Biological Abstracts <1990 to 2008>

1 (effectiveness or efficacy or immunogen* or impact* or reduction or durability or duration or "protective effect" or "real-world" or "real world").ab,ti. 1035809

2 (reduc* or effect*).ti. 612881

3 (vaccin* or immunis* or immuniz* or prophyla* or Immunoprophylaxis or "Immuno prophylaxis").ti. 48838

4 ("Hepatitis A" or "type A Hepatitis" or "Hepatitis type A").ab,ti. 1163

5 Hepatitis A Vaccines.kw,sh. 332

6 Viral Hepatitis Vaccines.fx,hw,kw,sh,xm,xs. 325

7 ("Hepatitis A" or "Hepatitis A virus" or "Hepatitis A Virus, Human").fx,hw,kw,sh,xm,xs. 2443

8 (Vaccines or Vaccination or Immunization or "Post-Exposure Prophylaxis" or "Pre-Exposure Prophylaxis" or "Immunization Programs" or "Primary Prevention").fx,hw,kw,sh,xm,xs.28734

9 Vaccine Efficacy.fx,hw,kw,sh,xm,xs. 174

10 1 or 2 1382586

11 3 or 8 57562

12 9 or 10 or 11 1404426

13 4 or 7 3017

14 12 and 13 2085

18 limit 14 to yr="2000 - 2008" 558

19 limit 14 to yr="1990 - 1999" 454

Biological Abstracts <1987 to 1989>

65 12 and 13

Biological Abstracts <2010 to 2012>

195 12 and 13

MEDLINE and EMBASE Databases within CAS STNext

FILE 'MEDLINE' ENTERED AT 11:45:07 ON 02 MAR 2026

L1 QUE "HEPATITIS A VACCINES"/CT OR "HEPATITIS A: PC, PREVENTION & CONTROL"/CT OR "HEPATITIS A: IM, IMMUNOLOGY"/CT

L2   QUE "HEPATITIS A VIRUS: PC, PREVENTION & CONTROL"/CT OR "HEPATITIS A VIRUS, HUMAN: PC, PREVENTION & CONTROL"/CT OR "HEPATITIS A VIRUS: IM, IMMUNOLOGY"/CT OR "HEPATITIS A VIRUS, HUMAN: IM, IMMUNOLOGY"/CT
L3   QUE L1 OR L2
L4   QUE ?VACCIN? OR ?IMMUNIS? OR ?IMMUNIZ? OR ?PROPHYLA?
L5   QUE "VACCINATION"/CT OR "VACCINE"/CT OR "VACCINES"/CT OR "VIRAL VACCINES"/CT OR "IMMUNIZATION"/CT OR "IMMUNIZATION PROGRAMS"/CT OR "IMMUNIZATION, SECONDARY"/CT
L6   QUE "MASS VACCINATION"/CT OR "PRIMARY PREVENTION"/CT OR "POST-EXPOSURE PROPHYLAXIS"/CT OR "PRE-EXPOSURE PROPHYLAXIS"/CT OR "VACCINE"/CT
L7   QUE L4 OR L5 OR L6
L8   QUE "HEPATITIS A"/CT OR "HEPATITIS A VIRUS"/CT OR "HEPATITIS A VIRUS, HUMAN"/CT
L9   QUE (HEPATITIS(2A)A)/BI OR HAV/TI,AB
L10 QUE L8 OR L9
L11 QUE "COMPARATIVE EFFECTIVENESS RESEARCH"/CT OR "VACCINE EFFICACY"/CT OR "TREATMENT OUTCOME"/CT
L12 QUE EFFECTIVNESS OR REAL(W)WORLD OR EFFICACY OR PROTECT?(2A)EFFECT?
L13 QUE (EFFECT? OR IMPACT? OR OUTCOME OR REDUCTION OR DURABL? OR DURABIL? OR DURATION OR REAL)(8A)L4
L14 QUE EFFECT?/TI OR IMPACT?/TI OR OUTCOME/TI OR (REAL/TI(W)WORLD/TI) OR DURABL?/TI OR DURABIL?/TI OR DURATION/TI OR INCIDENCE/TI(W)RATE/TI(W)RATIO/TI OR IRR/TI
L15 QUE L11 OR L12 OR L13 OR L14
L16   2566 SEA ((L3 OR (L7 AND L10)) AND L15) NOT ANIMAL/CT
D AN, DN, TRIAL, CS, CY, JT, ISN, DT, LA, PY TOT

FILE 'EMBASE' ENTERED AT 05:48:56 ON 02 MAR 2026
L17 QUE "HEPATITIS A: PC, PREVENTION"/CT OR "HEPATITIS A VACCINE"/CT
L18 QUE "HEPATITIS VACCINE"/CT OR "HEPATITIS: PC, PREVENTION"/CT OR "ACUTE HEPATITIS: PC, PREVENTION"/CT OR "VIRUS HEPATITIS: PC, PREVENTION"/CT
L19 QUE "ACUTE HEPATITIS"/CT OR HEPATITIS/CT OR "VIRUS HEPATITIS"/CT
L20 QUE "HEPATITIS A"/CT OR "ACUTE HEPATITIS A"/CT OR "HEPATITIS A VIRUS"/CT OR "ACUTE HEPATITIS A: PC, PREVENTION"/CT OR "HEPATITIS A VIRUS: PC, PREVENTION"/CT
L21 QUE "VACCINE"/CT OR "VACCINATION"/CT OR "IMMUNIZATION"/CT OR "MASS IMMUNIZATION"/CT OR "RNA IMMUNIZATION"/CT OR "PROPHYLAXIS"/CT
L22 QUE L17 OR (L18 AND (L20 OR L9)) OR (L19 AND (L20 OR L9) AND (L21 OR L4))
L23 QUE "CLINICAL EFFECTIVENESS"/CT OR "COMPARATIVE EFFECTIVENESS"/CT OR "DRUG EFFECT"/CT OR "PROGRAM EFFECTIVENESS"/CT
L24 QUE "VACCINE EFFECTIVENESS"/ST OR EFFECTIVENESS/ST OR "REAL-WORLD"/ST OR POST VACCINE SURVEILLANCE/ST OR "OUTCOME ASSESSMENT"/CT
L25   387 SEA L22 AND (L23 OR L24) AND HUMAN/CT
L26 QUE "HEPATITIS A: PC, PREVENTION"/CT OR "HEPATITIS A VACCINE"/CT OR "ACUTE HEPATITIS A: PC, PREVENTION"/CT OR "HEPATITIS A VIRUS: PC, PREVENTION"/CT
L27   2288 SEA L26 AND (L23 OR L24 OR L12 OR L13 OR L14) AND HUMAN/CT

FILE 'MEDLINE, EMBASE' ENTERED AT 05:55:55 ON 02 MAR 2026

L28 4012 DUP REM L16 L27 L25 (1229 DUPLICATES REMOVED)
ANSWERS '1-2565' FROM FILE MEDLINE
ANSWERS '2566-4012' FROM FILE EMBASE

FILE 'EMBASE' ENTERED AT 11:56:13 ON 02 MAR 2026
L31 1445 SEA (L29 OR L30)
D L31 TRIAL TOT

S-Table 2. Leave-one-out sensitivity analysis of pooled vaccine effectiveness estimates for pre-exposure and post-exposure hepatitis A vaccination

Vaccination	Omitted study	Pooled VE (95% CI)
**Pre-exposure**	Riedemann, 1992	95·7 (93·0–97·4)
	Werzberger, 1992	95·6 (92·9–97·2)
	Innis, 1994	95·9 (92·9–97·6)
	Richtmann, 1996	95·6 (92·9–97·3)
	Gong, 2003	95·6 (92·4–97·5)
	Mayorga Pérez, 2003	95·6 (92·9–97·3)
	Mao, 1997	95·6 (93·0–97·2)
	Zhao, 2000	95·9 (92·9–97·7)
	Averhoff, 2001	95·6 (92·7–97·4)
	Xu ZY, 2002	95·9 (93·0–97·6)
	Xu ZY, 2002	96·0 (92·9–97·7)
	Fisenka, 2008	95·9 (92·9–97·6)
	Mayorga, 2016	95·6 (92·6–97·3)
	Lin, 2018	95·9 (92·7–97·7)
	Im, 2020	96·3 (94·5–97·5)
**Post-exposure**	Príkazský, 1994	97·2 (89·0–99·3)
	McMahon, 1996	97·9 (91·8–99·5)
	Sagliocca, 1999	97·8 (91·4–99·5)
	Selnikova, 2008	96·8 (87·8–99·2)
	Shen, 2008	94·3 (82·2–98·2)
	Shen, 2008	96·6 (88·2–99·0)
	Freeman, 2014	97·1 (88·8–99·3)
	Parrón, 2017	97·1 (86·2–99·4)

VE, vaccine effectiveness; CI, confidence interval.
Note: Each row shows the pooled vaccine effectiveness estimate after sequential exclusion of the indicated study.

S-Table 3. Assessment of certainty of evidence for pooled hepatitis A vaccine effectiveness estimates.

Vaccination	Subgroup	Age group	Fixed-effect VE (95% CI)	Small-study effect	Egger-extrapolated VE at SE=0 (95% CI)	Imputed studies, n	Limitations signal, %	Inconsistency signal, %	Indirectness signal, %	Imprecision signal, %	Publication bias	Upgrade for large effect
**Pre-exposure**	Overall	Any	95·5 (93·5–96·9)	Yes	89·2 (78·3–94·6)	5	17·3	29·9	43·6	4·3	Not serious	Yes
	Inactivated vaccine	<18 years	97·1 (92·7–98·8)	Yes	87·3 (29·1–97·7)	4	17·1	4·3	43·8	5·0	Not serious	Yes
	Live vaccine	<18 years	96·0 (91·5–98·1)	No	—	1	23·6	0·0	41·6	4·4	Not serious	Yes
	RCTs	<18 years	97·0 (92·4–98·8)	No	—	3	2·2	20·1	0·0	6·3	Not serious	No
	Cohort studies	<18 years	95·9 (91·9–97·9)	No	—	1	33·3	0·0	67·4	4·6	Not serious	Yes
**Post-exposure**	Overall	Any	92·0 (88·9–94·3)	No	—	0	85·2	89·7	54·5	9·8	Not serious	No
	Close-contact setting	Any	97·1 (93·9–98·6)	No	—	1	69·2	67·0	2·4	19·5	Not serious	No
	Outbreak-area setting	Any	85·8 (75·2–91·9)	No	—	0	100·0	89·7	100·0	20·6	Serious	No

Fixed-effect VE was estimated using the inverse-variance model. Small-study effects were assessed using meta-regression of the effect size on its standard error. In this model, the coefficient for the standard error was interpreted as the bias coefficient. Evidence of a small-study effect was considered present when the bias coefficient was statistically significantly different from zero ($p < 0.05$). Accordingly, “Yes” indicates evidence of a small-study effect, whereas “No” indicates no evidence of a small-study effect ($p \geq 0.05$). When evidence of a small-study effect was present, vaccine effectiveness was additionally extrapolated from the meta-regression model at SE = 0, representing the expected estimate in a hypothetical infinitely precise study. Imputed studies were estimated using the trim-and-fill method. Signal values represent the weighted proportion of contributing evidence judged as problematic for the corresponding GRADE domain. Publication bias was judged from fixed-effect versus random-effects comparisons, trim-and-fill analyses, and Egger regression for small-study effects. VE=vaccine effectiveness. SE=standard error. RCT=randomised controlled trial.

S-Table 4. Ecological studies evaluating the impact of hepatitis A vaccination on incidence in target populations.

Author, year	Country	Target population	Vaccine (doses)	Pre period	Pre IR (/100,000 PY)	Post period	Post IR (/100,000 PY)	Vaccination coverage (%)	VI (%)	Notes
**Vacchino, 2008**[1]	Argentina	1 year	Inactivated (1)	1998–2002	67·9	2007	11·5	95	83	Universal 1-dose programme from June 2005 at age 12 months; brand unspecified
**Vizzotti, 2014**[2]	Argentina	<4 years	Inactivated (1)	2000–2002	4·0	2009–2011	0·5	75–80	88	
**Hanna, 2004**[3]	Australia	<5 years	Inactivated (≥1)	1996–1999	149·0	2000–2003	3·5	80–85	98	Free 2-dose programme from February 1999 for Indigenous children in Queensland, with catch-up to age 6; brand unspecified
**Thompson, 2017**[4]	Australia	<5 years	Inactivated (≥1)	2000–2005	5·0	2006–2014	1·1	35–45	78	Free 2-dose programme from November 2005 for Indigenous children and from July 2013 for non-Indigenous children in selected jurisdictions (Queensland, South Australia, Western Australia, and the Northern Territory);; combined IRs; brand unspecified
**Fisenka, 2008**[5]	Belarus	6–9 years	Inactivated (2)	2000–2002	8·2	2005–2006	0·0	75–85	100	Universal 2-dose programme from 2003 at school entry in Minsk; mainly Avaxim
**Souto, 2019**[6]	Brazil	<5 years	Inactivated (1)	2010–2013	6·4	2015–2017	1·1	35–40	82	Universal 1-dose programme from July-August 2014 at ages 15–24 months; catch-up expanded in 2017; Vaqta
**Bierrenbach, 2021**[7]	Brazil	1–4 years	Inactivated (1)	2010–2013	6·0	2015–2018	0·7	45–55	88	
**Sun, 2018**[8]	China	1–4 years	Inactivated (2)	2004–2007	0·6	2012–2016	0·2	72–75	73	Nationwide EPI from 2008: 1-dose live schedule at 18 months in 27 regions and 2-dose inactivated schedule at 18 and 24 months in 4 regions
			Live (1)	2004–2007	10·0	2008–2016	3·7	65–75	63	
**Mao, 1997**[9]	China	1–15 years	Live (1)	1983–1990	213·6	1991–1995	8·4	85–89	96	Large-scale 1-dose programme from 1991 at ages 1–15 years in Jiaojiang City; H2 live vaccine·
**Ming, 2024**[10]	China	2–9 years	Inactivated/live (≥1)	2005–2007	13·0	2008–2019	3·4	45–55	81	Nationwide EPI from 2008: 1-dose live schedule at 18 months in 27 regions and 2-dose inactivated schedule at 18 and 24 months in 4 regions

Author, year	Country	Target population	Vaccine (doses)	Pre period	Pre IR (/100,000 PY)	Post period	Post IR (/100,000 PY)	Vaccination coverage (%)	VI (%)	Notes
**Dagan, 2005**[11]	Israel	1–4 years	Inactivated (2)	1993–1998	128·9	2002–2004	2·3	70–80	98	Universal 2-dose programme from July 1999 at ages 18–24 months; Havrix
**Chodick, 2008**[12]	Israel	<5 years	Inactivated (2)	1998	239·4	2007	2·2	89	99	
**Levine, 2015**[13]	Israel	1–4 years	Inactivated (≥1)	1993–1998	155·0	2003–2012	2·0	88–92	99	Universal 2-dose programme from July 1999 at ages 18–24 months; brand unspecified
		5–9 years			210·0	2008–2012	1·0	60–65	100	
**Estripeaut, 2015**[14]	Panama	1–4 years	Inactivated (≥1)	2000–2006	63·8	2008–2010	7·0	55–65	89	Universal 2-dose programme from April 2007 at ages 12–18 months; Havrix
**Domínguez, 2003**[15]	Spain	10–14 years	Inactivated (≥1)	1996–1998	10·3	1999–2001	1·8	50–55	83	Universal programme from September 1998 at age 12 years in Catalonia; Twinrix·
**Tsou, 2011**[16]	Taiwan	<9 years	Inactivated (≥1)	1995	240·6	2003–2008	0·3	70–75	100	Routine 2- or 3-dose vaccination from June 1995 at ages 15 months–12 years in high-risk townships; Havrix
**Singleton, 2010**[17]	USA	<14 years	Inactivated (≥1)	1972–1995	112·4	2002–2007	0·2	70–80	100	Universal 2-dose vaccination from 1996 at ages 2–14 years in Alaska; expanded to 2–18 years in 1997 and 1–18 years in 2006; brand unspecified
**Erhart, 2012**[18]	USA	1–4 years	Inactivated (≥1)	1994–1995	81·0	2006–2007	0·6	60–65	99	Free 2- or 3-dose vaccination from 1996 at ages 2–12 years in Arizona; expanded statewide to ≥2 years in 1999; brand unspecified
		5–9 years			146·0		1·7	75–80	99	
**Averhoff, 2001**[19]	USA	<17 years	Inactivated (≥1)	1990–1994	67·3	1995–2000	13·5	35–66	80	Routine 2-dose vaccination from January 1995 at ages 2–12 years in Butte County, California; expanded to ages 2–17 years in 2000; Vaqta

Pre IR and Post IR are incidence rates before and after vaccination, respectively, expressed per 100,000 person-years. Vaccination rate indicates estimated coverage in the target population during the post-vaccination period; VI (%), vaccine impact, was calculated as 1 − (post-vaccination IR / pre-vaccination IR) × 100%

1 Vacchino MN. Incidence of Hepatitis A in Argentina after vaccination. *J Viral Hepat* 2008; **15**: 47–50.
2 Vizzotti C, González J, Gentile A, *et al.* Impact of the Single-dose Immunization Strategy Against Hepatitis A in Argentina. *Pediatr Infect Dis J* 2014; **33**: 84–8.
3 Hanna JN, Hills SL, Humphreys JL. Impact of hepatitis A vaccination of Indigenous children on notifications of hepatitis A in north Queensland. *Med J Aust* 2004; **181**: 482–5.
4 Thompson C, Dey A, Fearnley E, Polkinghorne B, Beard F. Impact of the national targeted Hepatitis A immunisation program in Australia: 2000–2014. *Vaccine* 2017; **35**: 170–6.
5 Fisenka EG, Germanovich FA, Glinskaya IN, Lyabis OI, Rasuli AM. Effectiveness of universal hepatitis A immunization of children in Minsk City, Belarus: four-year follow-up. *J Viral Hepat* 2008; **15**: 57–61.
6 Souto FJD, De Brito WI, Fontes CJF. Impact of the single-dose universal mass vaccination strategy against hepatitis A in Brazil. *Vaccine* 2019; **37**: 771–5.
7 Bierrenbach AL, Choi Y, Batista PDM, *et al.* The Impact of an Inactivated Hepatitis A Vaccine with One Dose in Brazil: A Retrospective Time-Series. *Vaccines* 2021; **9**: 407.
8 Sun X, Wang F, Zheng H, *et al.* The impact of expanded program on immunization with live attenuated and inactivated Hepatitis A vaccines in China, 2004–2016. *Vaccine* 2018; **36**: 1279–84.
9 Mao JS, Chai SA, Xie RY, *et al.* Further evaluation of the safety and protective efficacy of live attenuated hepatitis A vaccine (H2-strain) in humans. *Vaccine* 1997; **15**: 944–7.
10 Ming B-W, Li L, Huang H-N, *et al.* The Effectiveness of National Expanded Program on Immunization With Hepatitis A Vaccines in the Chinese Mainland: Interrupted Time-Series Analysis. *JMIR Public Health Surveill* 2024; **10**: e53982.
11 Dagan R. Incidence of Hepatitis A in Israel Following Universal Immunization of Toddlers. *JAMA* 2005; **294**: 202.
12 Chodick G, Heymann AD, Ashkenazi S, Kokia E, Shalev V. Long-term trends in hepatitis A incidence following the inclusion of Hepatitis A vaccine in the routine nationwide immunization program. *J Viral Hepat* 2008; **15**: 62–5.
13 Levine H, Kopel E, Anis E, Givon-Lavi N, Dagan R. The impact of a national routine immunisation programme initiated in 1999 on Hepatitis A incidence in Israel, 1993 to 2012. *Eurosurveillance* 2015; **20**. DOI:10.2807/1560-7917.ES2015.20.7.21040.
14 Estripeaut D, Contreras R, Tinajeros O, *et al.* Impact of Hepatitis A vaccination with a two-dose schedule in Panama: Results of epidemiological surveillance and time trend analysis. *Vaccine* 2015; **33**: 3200–7.
15 Domínguez A. Effectiveness of a mass hepatitis A vaccination program in preadolescents. *Vaccine* 2003; **21**: 698–701.
16 Tsou T-P, Liu C-C, Huang J-J, Tsai K-J, Chang H-F. Change in hepatitis A epidemiology after vaccinating high risk children in Taiwan, 1995–2008. *Vaccine* 2011; **29**: 2956–61.
17 Singleton RJ, Hess S, Bulkow LR, Castrodale L, Provo G, McMahon BJ. Impact of a statewide childhood vaccine program in controlling hepatitis A virus infections in Alaska. *Vaccine* 2010; **28**: 6298–304.
18 Erhart LM, Ernst KC. The changing epidemiology of hepatitis A in Arizona following intensive immunization programs (1988–2007). *Vaccine* 2012; **30**: 6103–10.
19 Averhoff F. Control of Hepatitis A Through Routine Vaccination of Children. *JAMA* 2001; **286**: 2968.

S-Figure 1. Summary of RoB 2 judgments across included randomised controlled trials

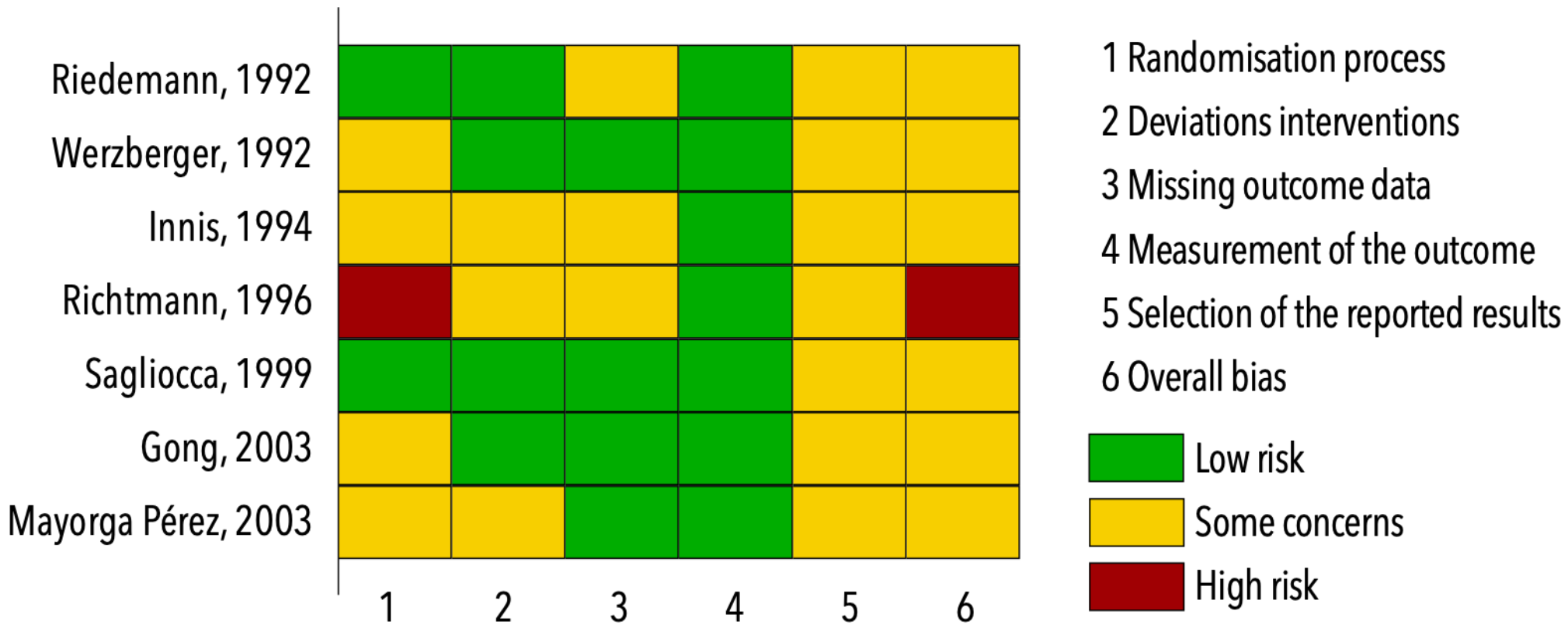

Summary of RoB 2 judgments across included randomised controlled trials. Each cell represents the judgement for one RoB 2 domain within an individual trial. Green indicates low risk of bias, yellow indicates some concerns, and red indicates high risk of bias.

S-Figure 2. Summary of ROBINS-I judgments across included cohort studies

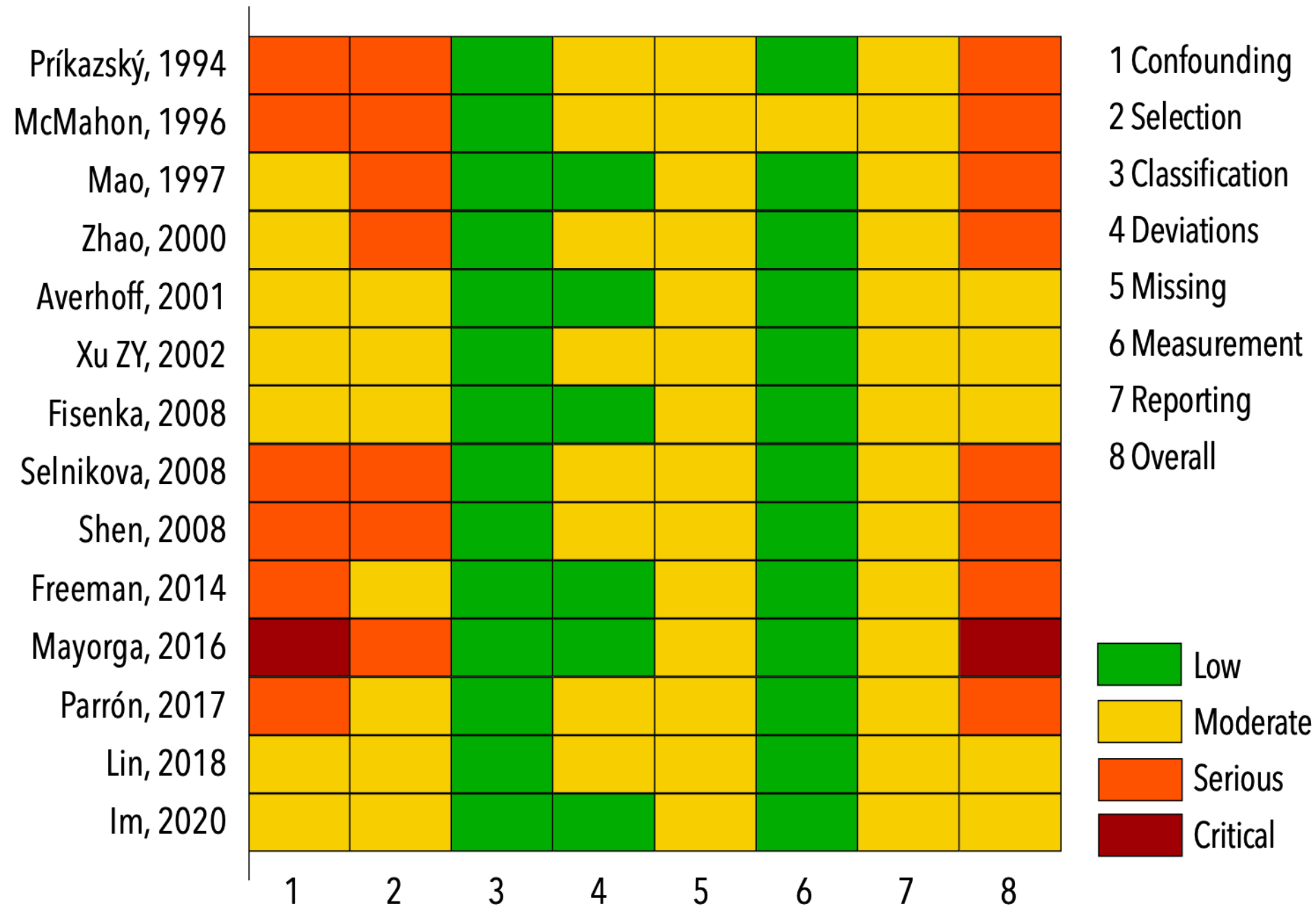

Summary of ROBINS-I judgments across included cohort studies. Each cell represents the judgement for one ROBINS-I domain within an individual study. Green indicates low risk of bias, yellow indicates moderate risk, orange indicates serious risk, and red indicates critical risk of bias.

S-Figure 3. Funnel plot for pre-exposure hepatitis A vaccination.

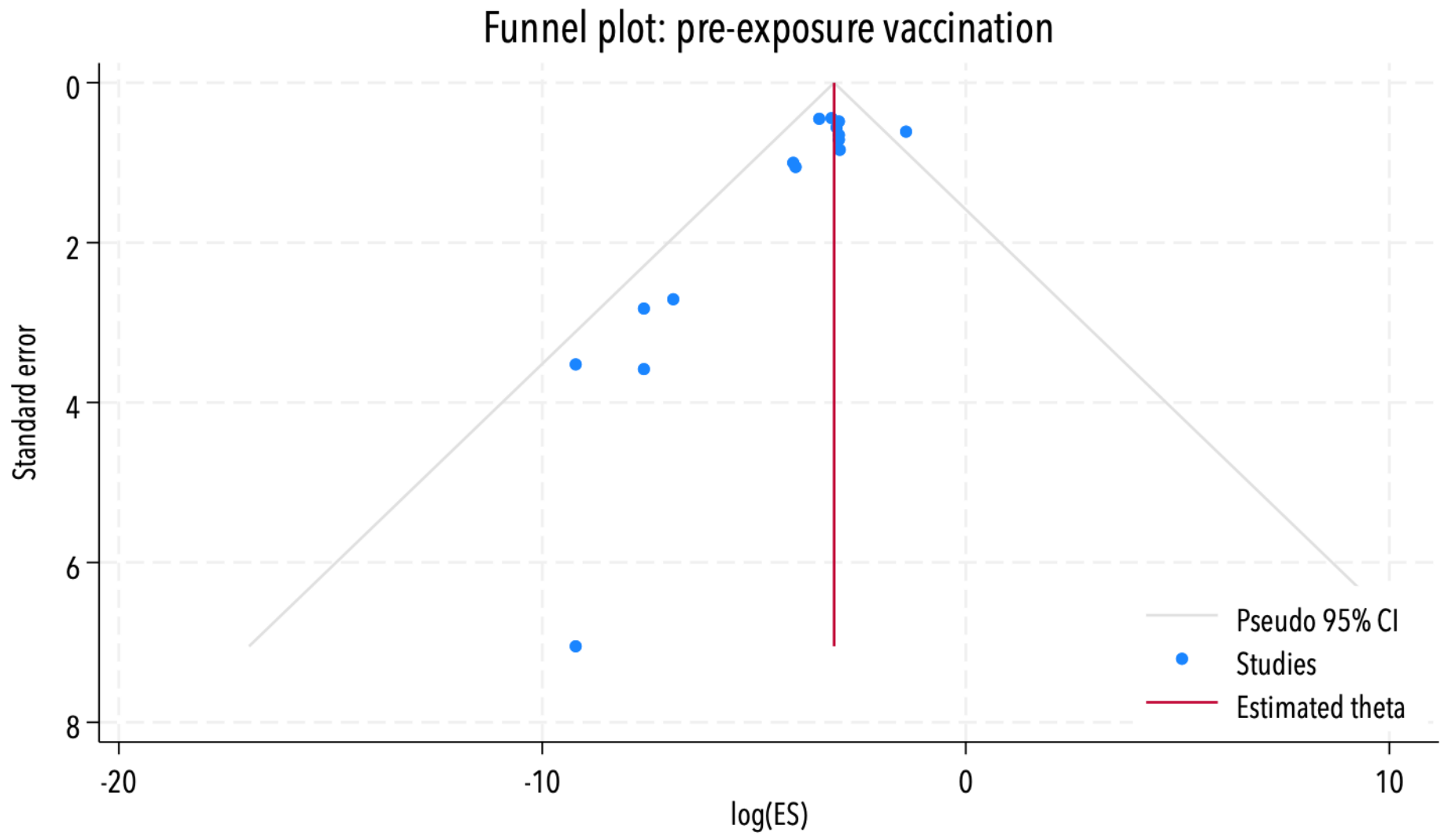

Funnel plot showing the relationship between the log-transformed effect size [log(ES)] and its standard error for studies evaluating pre-exposure hepatitis A vaccination. Each point represents an individual study estimate. The vertical line indicates the pooled effect estimate, and the diagonal lines represent the pseudo 95% confidence limits. The plot suggests some asymmetry, with smaller and less precise studies tending to report larger vaccine effects. However, the influence of small-study effects was considered limited because the fixed-effect estimate, trim-and-fill adjusted estimate, and meta-regression extrapolated estimate differed from the primary pooled estimate by less than 10 percentage points. Blue circles: individual studies; red vertical line: pooled estimated effect; grey diagonal lines: pseudo 95% confidence limits.

S-Figure 4. Funnel plot for post-exposure hepatitis A vaccination.

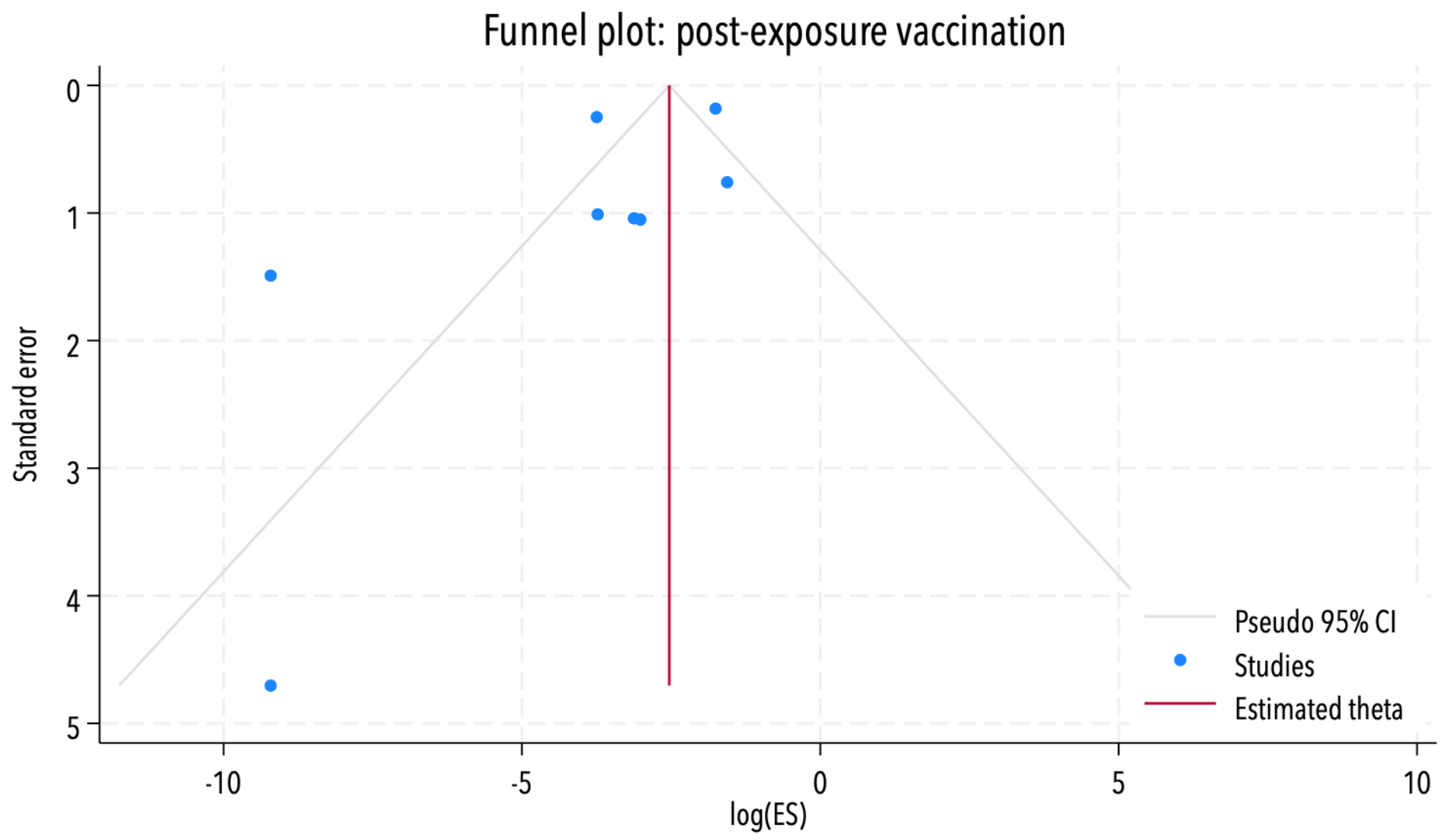

Funnel plot showing the relationship between the log-transformed effect size [log(ES)] and its standard error for studies evaluating post-exposure hepatitis A vaccination. Each point represents an individual study estimate. The vertical line indicates the pooled effect estimate, and the diagonal lines represent the pseudo 95% confidence limits. The plot suggests possible asymmetry, with smaller and less precise studies tending to report larger vaccine effects. This was taken into account in the assessment of small-study effects and publication bias. Blue circles: individual studies; red vertical line: pooled estimated effect; grey diagonal lines: pseudo 95% confidence limits.

S-Figure 5. Factors associated with differences in hepatitis A vaccine impact in paediatric target populations

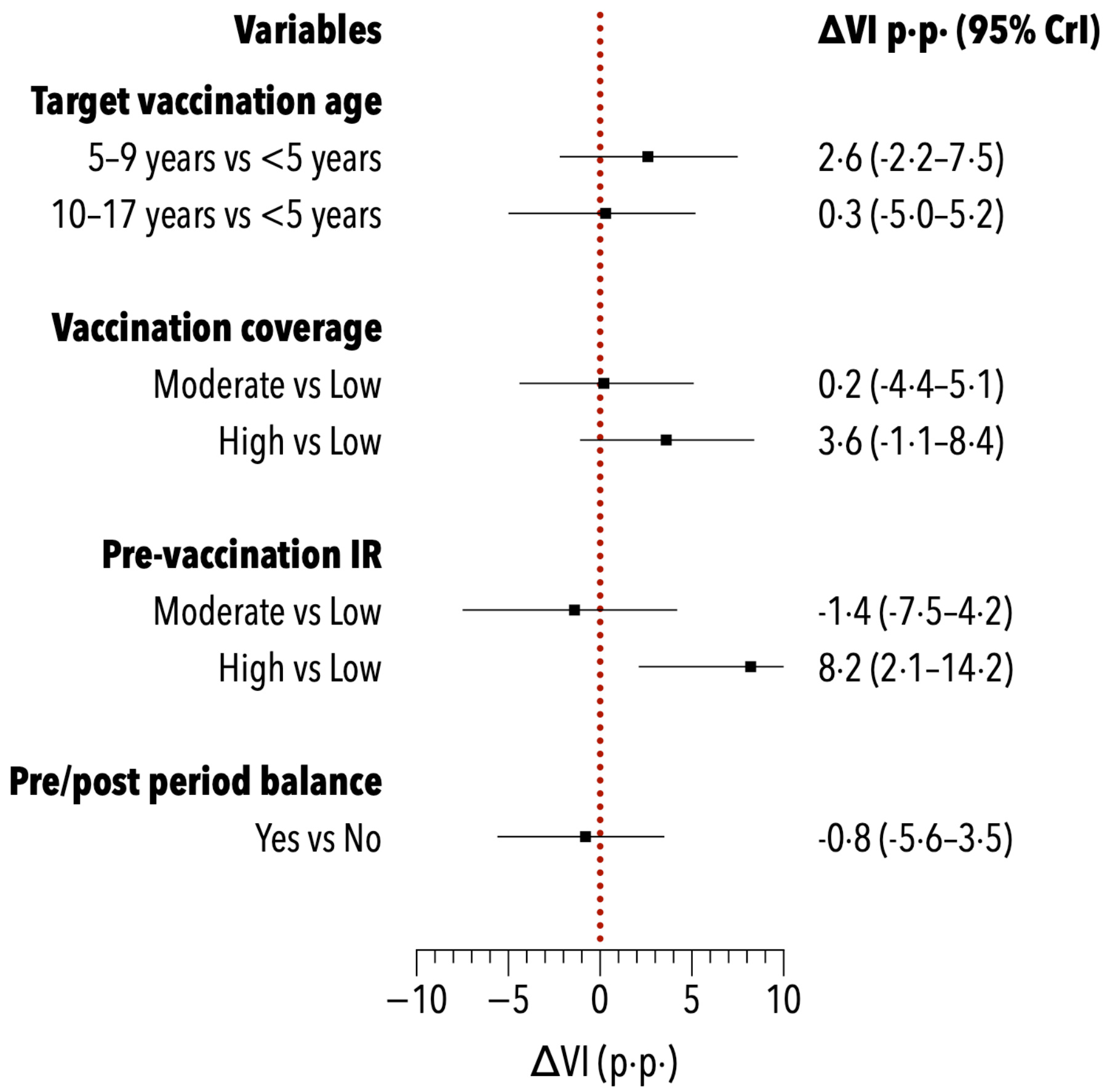

Factors associated with differences in hepatitis A vaccine impact in paediatric target populations. Squares represent posterior mean differences in vaccine impact (ΔVI), and horizontal lines indicate 95% credible intervals (CrI) from the Bayesian regression model. Estimates are expressed in percentage points (p·p·) relative to the reference categories: target vaccination age <5 years, low vaccination coverage, low pre-vaccination incidence rate (IR; <10 cases per 100,000 person-years), and balanced pre-/post-vaccination periods. Pre-vaccination IR was classified as low (<10), moderate (10–100), and high (>100 cases per 100 000 person-years). Positive values indicate higher vaccine impact compared with the reference category.
